# Advanced Photoelectrochemical Hydrogen Generation by CdO-g-C_3_N_4_ in Aqueous Medium under Visible Light

**DOI:** 10.3390/molecules27248646

**Published:** 2022-12-07

**Authors:** Naseer Iqbal, Muhammad Shahzeb Khan, Muhammad Zubair, Safyan Akram Khan, Asghar Ali, Naif Aldhafeeri, Saud Alsahli, Misheal Alanzi, Abdelazeez Enazi, Talal Alroyle, Abdullatif Alrashidi

**Affiliations:** 1 Department of Chemistry, College of Science, University of Hafr Al Batin, P.O. Box 1803, Hafr Al Batin 39524, Saudi Arabia; 2 Department of Mechanical Engineering, College of Engineering, University of Hafr Al Batin, P.O. Box 1803, Hafr Al Batin 39524, Saudi Arabia; 3 Department of Physics, College of Science, University of Hafr Al Batin, P.O. Box 1803, Hafr Al Batin 39524, Saudi Arabia; 4 Interdisciplinary Research Center for Hydrogen & Energy Storage (IRC-HES), King Fahd University of Petroleum & Minerals (KFUPM), P.O. Box 5040, Dhahran 31261, Saudi Arabia

**Keywords:** photocatalysis, water-splitting, CdO nanorods, carbon nitride, photoelectrochemical

## Abstract

Herein, hydrothermal fabrication of CdO-g-C_3_N_4_ photocatalyst for a substantially better photocatalytic recital in water splitting is presented. The XRD analysis confirms the cubic phase of CdO-g-C_3_N_4_, whereas FTIR and UV-VIS studies revealed the presence of respective groups and a median band gap energy (2.55 eV) of the photocatalyst, respectively, which further enhanced its photo-electrochemical (PEC) properties. The SEM displays the oblong structures of g-C_3_N_4_ sheets and nano rod-like morphology of CdO and CdO-g-C_3_N_4_, respectively. The HR-TEM exhibits morphology & orientation of the grains and substantiates the polycrystal-line nature of CdO-g-C_3_N_4_ nanocomposite. The photocatalytic water-splitting concert is evaluated by PEC experiments under 1 SUN visible light irradiation. Linear sweep voltammetry (LSV), chronoamperometry (CA), and electrochemical impedance spectroscopy (EIS) comprehend the CdO-g-C_3_N_4_ as a hydrogen evolution photocatalyst. A photocurrent density beyond ≥5 mA/cm^2^ is recorded from CdO-g-C_3_N_4_, which is 5–6 folds greater than pure CdO and g-C_3_N_4_. The efficient separation and transfer of charges allocated to CdO-g-C_3_N_4_ and fabricating heterojunctions between g-C_3_N_4_ and CdO suppresses the unfavorable electron-hole pairs recombination process. Thus, it recesses charge transfer resistance, augmenting enhanced photocatalytic performance under 1 SUN irradiation.

## 1. Introduction

Semiconducting nanomaterials for photoelectrochemical water splitting earned substantial consideration in recent decades. This is imperative to fulfill the intensifying global energy challenges and to meet the renewable energy demand [1,2,3,4,5,6,7,8,9,10]. Solar energy is an important renewable energy source that can split water into molecular hydrogen and oxygen with the aid of photocatalysts [11,12]. Solar energy is the most abundant and promising source of sustainable H_2_ production. One of the key challenges is its proper utilization to develop innovative systems that can use this renewable energy source effectively as an energy intake to drive the reactions for producing H_2_ in an environmentally friendly way. Hydrogen (H_2_), the future fuel, is a resourceful energy carrier. It is among the promising sources of clean energy to confront several energy challenges. H_2_ bears remarkable properties that makes it a potential source of energy. H_2_ is abundantly found in combination with other elements such as oxygen, carbon, and nitrogen. 75% of the universe’s mass contains H_2_. It is a colorless, odorless gas that accounts for higher energy source with zero greenhouse emissions, high gravimetric energy density, low heating value of ∼120 MJ kg^−1^, etc. [13,14,15]. Since the last few decades, hydrogen has been used majorly in the synthesis of methanol, ammonia and in petroleum refinement. Furthermore, cosmonauts use hydrogen as fuel for their space vehicles and in fuel cells that provide heat, electricity and drinking water in space. To utilize hydrogen, it must be separated from other substances. Fuel cells are one of the stratagems by which hydrogen can be directly converted into electricity. In the near future, hydrogen could be used to fuel automobiles and aircraft and to provide power to fulfill domestic and commercial needs. Furthermore, H_2_ can be employed in current industrial setups that are being used for conventional fuels. Therefore, lately several countries have launched sustainable energy strategies and programs to nurture the implementation of H_2_ based technologies on various fronts, which will help in instituting a low carbon environment [13,15].

Photoelectrochemical water splitting consists of an oxidation/reduction of half-cell reactions—i.e., four-proton, four-electron process—which requires large over-potential in order to restrain the competing electron-hole recombination processes at the expense of solar energy [11,12,16,17,18,19]. Photoelectrochemical solar water splitting for H_2_ and O_2_ production involves an appropriate material with substantial band gap energy that could assist the electron holes’ mobility in an effective manner. Usually, semiconductors with a bandgap below 3 eV are considered efficient for solar driven water splitting [16,17,18]. For an effective PEC process, the photocatalysts and the PEC system should be efficient, stable, durable under harsh experimental or operational conditions and result in minimal or no environmental impacts. It should also be capable of absorbing a large fraction of the visible light and retaining well-aligned band energy for thermodynamically promising charge transfer reactions. Above all such systems should be cost-effective [19,20,21]. To comprehend all the aforementioned characteristics required for effectual solar water splitting with significant solar-to-chemical energy conversion and in order to make these systems commercially viable, significant research efforts are in progress [22,23,24,25,26]. Nevertheless, there are enough opportunities for finding superior, inexpensive, and effective photocatalyts.

Several transition-metal oxides have been widely reported due to their intriguing electrical, optical and catalytic properties [27,28,29,30,31,32]. Cadmium oxide (CdO) is among one of the nanomaterials which is an n-type semiconductor with a cubic (FCC) crystal structure and owes many imperative properties such as wide band-gap (2.16–2.6 eV) and refractive index (n = 2.75), high density (8150 kg/m^3^), low resistance and high transmittance in the visible range [33,34]. Based on these properties, CdO is used in solar cells, phototransistors, catalysts, transparent electrodes, gas sensors, photodiodes, photoelectric components [34,35,36,37], etc. However, its photoelectrochemical properties are not fully explored, especially in solar driven water splitting. It is worth mentioning here that CdS and CdSe are also known light absorption photocatalysts but severe photo corrosion due to sulfur leaching or Se degradation greatly affect its practical stability in the PEC water splitting process. Furthermore, their toxicity also limits their large-scale application in industry [38]. Thus, oxygen incorporation is more suitable with cadmium as compared to S or Se in addition to the doping or intermixing of CdO with suitable semiconducting supporting materials such as graphitic Carbon Nitride (g-C_3_N_4_) to achieve maximal output.

The g-C_3_N_4_ has attracted widespread attention in recent years [39] as an emerging carbon based semiconductor. Its excellent features, such as suitable band gap (2.71 eV), easily tunable electronic structure and absorption of visible light (460nm wavelength) and high physical and chemical stability make it an attractive entrant in semiconducting industrial applications. g-C_3_N_4_ has two basic structural units: s-triazine (C_3_N_4_) and tris-s-triazine (C_6_N_7_) rings; for g-C_3_N_4_, the regular three s-triazine units structure is considered to be among the most stable phase studies by Density Functional Theory (DFT) [40] Despite several advantages, g-C_3_N_4_ also have shortcomings, such as high recombination rate for photogenerated electron/hole pairs, poor electron conductivity, slow hole- Shifts, etc., that limit its use as a photoelectrode material. The PEC performance of g-C_3_N_4_ can be improved by constructing different nanoscale morphologies of g-C_3_N_4_ [14,16,39] doping or physical mixing of different metals to produce heterogeneous photocatalysts that could result in improved PEC efficiency and visible light absorption. So, morphologically tuned g-C_3_N_4_ nanomaterials dopped or intermixed with metal or metal oxides could develop nanoscale heterogeneous nanomaterials with the potential for PEC water splitting [14,16,39] properties.

In Table 1, we compared several recent examples of Cd based g-C_3_N_4_ nanomaterials which are used in photoelectrochemical water splitting and in photodegradation applications. It is evident that ample of g-C_3_N_4_ materials impregnated or dopped with metal/metal oxides following various synthetic strategies are explored.

In the current research, we aimed at developing more stable, inexpensive and effective metal oxide based visible light-driven photocatalysts nanocomposites as photoanode materials to address the challenges in PEC water splitting. Thus, to enhance the PEC water splitting, we used CdO, graphitic Carbon Nitride (g-C_3_N_4_) and CdO-g-C_3_N_4_ nanocomposites. These photocatalytic materials were prepared by Ultrasonication, precipitation and hydrothermal methods. The nanomaterials were characterized by physicochemical analysis methods such as XRD, FTIR, UV-VIS, PL and SEM, HR-TEM for estimation of their structure property relationships, elemental composition, and morphological properties. For solar driven water splitting CdO, g-C_3_N_4_ and CdO-g-C_3_N_4_ photoanode materials are also evaluated for enhanced PEC properties by electrochemical methods under 1 SUN solar irradiation.

## 2. Results and Discussion

### 2.1. X-ray Diffraction (XRD) Analysis

**Figure 1:**
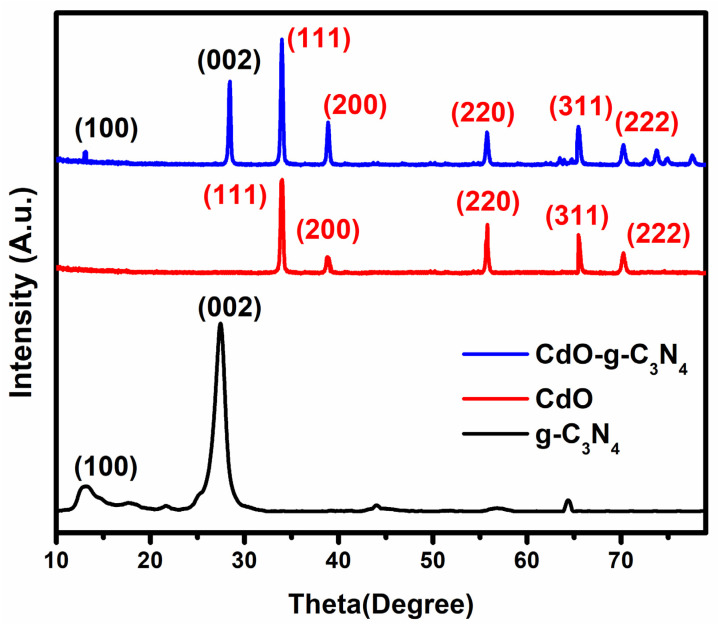
The XRD patterns of g-C_3_N_4_, CdO, and CdO-g-C_3_N_4_ nanomaterials.

The XRD diffraction spectrum of pristine g-C_3_N_4_, CdO, and the composite CdO-g-C_3_N_4_ are shown in Figure 1. In the comparison spectrum, all the developed materials appeared in their respective characteristic peaks and confirms their crystalline nature after undergoing the complete hydrothermal process.

The analysis was performed to assess the phase formation during the processing of pristine g-C_3_N_4_, CdO, and the composite CdO-g-C_3_N_4_, the diffraction patterns of each material are shown in Figure 1. The patterns confirmed the presence of g-C_3_N_4_, CdO, and CdO-g-C_3_N_4_ in their respective materials. The diffraction peaks in the spectrum for g-C_3_N_4_ were observed at Bragg angles 13° and 27° concerning their miller indices 100 & 002, and the peaks are in agreement with the JCPDS card numbers 87–1526 [39,40]. Furthermore, the XRD pattern of CdO exhibited the formation of cubic phase crystal structure and indexed the following miller indices (111), (200), (220), (311), (222) at the respective diffraction peaks at 2θ values. The observed diffraction peaks are well aligned with the JCPDS card # 05–0640 [38]. The XRD pattern of CdO-g-C_3_N_4_ nanomaterials also showed and confirmed the presence of all characteristic peaks of CdO and g-C_3_N_4_ at their respective 2θ values. It supports that the precipitation method has converted the cadmium nitrate into cadmium oxide and the hydrothermal method supports the CdO and g-C_3_N_4_ intermixing into a crystalline CdO-g-C_3_N_4_ nanomaterial. The XRD studies further revealed that in CdO-g-C_3_N_4_ the CdO crystallizes into the cubic Fm$\bar{3}$m space group and that each Cd^2+^ is bonded to six equivalent O^2−^ atoms to form a mixture of edge and corner-sharing CdO on the triazine rings of g-C_3_N_4_. All Cd–O bond lengths are 2.39 Å. O^2−^ and are bonded to six equivalent Cd^2+^ atoms to form a mixture of edge and corner-sharing OCd_6_ octahedra.

### 2.2. Scaning Electron Microscopy (SEM) and High Resolution Transmission Electron Microscopy (HR-TEM)

**Figure 2:**
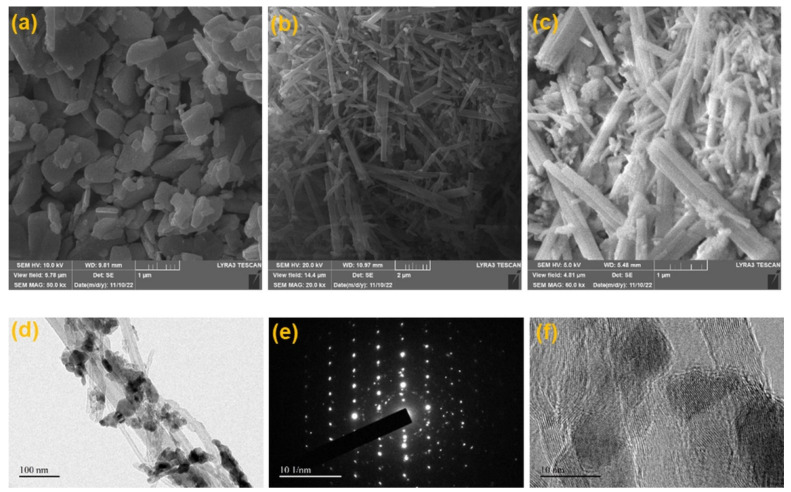
SEM and HR-TEM micrographs of (**a**) oblong shape g-C_3_N_4_ nanosheets (**b**) CdO nanorods indicating homogenous distribution and uniform thickness of 50−150 nm and 1–3 ±μm length (**c**) Nanorod-like structures of CdO-g-C_3_N_4_ photocatalyst. (**d**) H-RTEM micrograph of CdO-g-C_3_N_4_ nanocomposite. (**e**) SAED pattern of CdO-g-C_3_N_4_ composite nanorods. (**f**) Fringe spacing of polycrystalline CdO-g-C_3_N_4_ nanocomposite.

The topography and morphology of developed materials were investigated using an advanced scanning electron microscopic technique. Figure 2a shows the morphology of g-C_3_N_4,_ and the oblong or long-flattened grains show the nature of g-C_3_N_4_ to be nanosheets. In addition, Figure 2b presents the micrographs of CdO, and it can be observed that the CdO consists of nanorods morphology with an average thickness of 50–150 nm and 1–3 ± μm length. Furthermore, Figure 2c displays the structural nature of the CdO-g-C_3_N_4_ composite, and it is evident that a blend of both the entities—nanosheets and nanorods (50–150 nm and 1–3± μm length)—are present in the SEM micrograph. Since both the major ingredients, g-C_3_N_4_ and CdO, exhibited their sizes in the nanoscale, and due to their lower grain sizes, the chances of agglomeration are quite bright due to large surface affinity. Nevertheless, Figure 2c attests to the validity of the process and parameters to produce uniform and homogenized CdO-g-C_3_N_4_ composite nanorods which will be further put through photoelectrochemical (PEC) characterization.

The HR-TEM micrographs of CdO-g-C_3_N_4_ are demonstrated in Figure 2d–f. Nanorods like structure of the CdO can be observed in Figure 2d with some irregular shaped g-C_3_N_4_ nanosheets. The HR-TEM Figure 2d reveals that the CdO nanorods were grown from nanoscale crystals, and the visible gaps and holes in between the nanorods were developed due to detachment and further vaporization of H_2_O from the Cd (NO_3_)_2_·4H_2_O precursor, while CdO synthesis was followed by heat treatment in autoclave for 180 °C. In addition, the diameter of nanorods ranges from 50–60 nm with an average length of 2–3 μm which makes its surface area huge, making its surface more active for electron and holes interaction. Thus, producing CdO-g-C_3_N_4_ is a promising candidate for photoelectrochemical and photocatalytic applications. Figure 2e exhibits the selected area diffraction (SAED) pattern of a CdO-g-C_3_N_4_ nanocomposite which is polycrystalline in nature and crystals are placed at different orientations which can be deduced from the numerous bright dots available on the pattern. The concentric rings in the pattern are in good agreement with the XRD planes (111), (200), (220), (311) and (222) [44,49,50]. A higher magnification micrograph, as in Figure 2f, demonstrated the size and morphology of grains and nanorod shaped like structures. The different orientation of the lattices in various grains manifests the polycrystalline nature of the CdO-g-C_3_N_4_ and shows the lattice spacing of 0.2 to 0.3 nm in Figure 2f, which is conforming to the standard lattice spacing of 0.235 nm in CdO for (200) crystal plane [51,52].

### 2.3. FTIR-Spectroscopic Analysis

**Figure 3:**
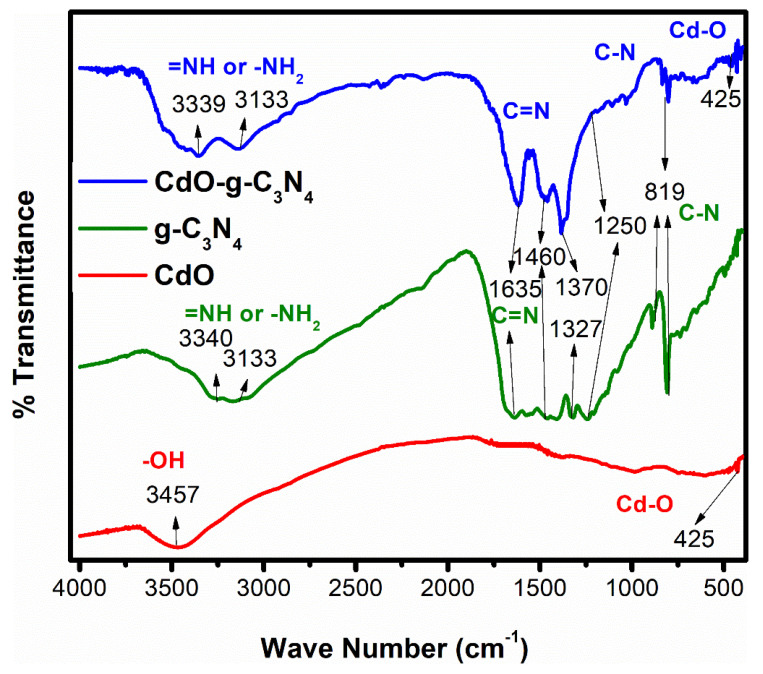
FTIR spectra of pure g-C_3_N_4_, CdO nanorods, and CdO-g-C_3_N_4_ photocatalysts.

To assess the chemical bonds and functional groups in the fabricated materials g-C_3_N_4_, CdO, and CdO-g-C_3_N_4,_ FTIR spectroscopy was performed within the range 370–4000 cm^−1^ as described in Figure 3. The presence of triazine structure in g-C_3_N_4_ nanosheets are validated by the emergence of characteristics heterocyclic ν (C–N/C = N) stretching vibrations and ν (N–H) shearing vibrations bands from 1250 to 1650 cm^−1^ and 3100–3600 cm^−1^ respectively. The large broad bands in g-C_3_N_4_ and CdO-g-C_3_N_4_ are the distinctive vibrational ν (C–N/C = N) bands of triazine units appeared at 12,501,327–70 cm^−1^ are assigned to stretching vibrations of C–NH–C, i.e., partially condensed, and C–N(–C)–C, i.e., fully condensed units, respectively. A strong peak at 819 cm^−1^ complemented the characteristic breathing mode vibrations of triazine units, which also reinforces growth of g-C_3_N_4_ [53]. The broad vibrational bands in g-C_3_N_4_ and Cdo-g-C_3_N_4_ from 3100 to 3600 cm^−1^ are assigned to free uncondensed terminal amino groups (-NH_2_ or =NH). 

As discussed earlier, the 1200–1700 cm^−1^ bands are arising due to skeletal C = N heterocycle stretches, consisting of trigonal (N-(C)_3_) and bridging C-NH-C units of the extended C-N-C network in the g-C_3_N_4_ structure [53]. The FTIR absorption bands recorded beneath ~ 1000 cm^−1^ are the fingerprint zone of metal oxides. Hence, the peak witnessed at 425 cm^−1^ in the FTIR spectrum of both the pristine CdO and CdO-g-C_3_N_4_ composite is referred to typical vibrational mode of Cd-O linkage, and it is well aligned with the reported results [54]. The other peak at 3457 cm^−1^ corresponds to hydroxyl group (-OH) due to moisture. In addition, the FTIR characterization of the CdO, g-C_3_N_4_ & CdO-g-C_3_N_4_ confirms its unadulteration by providing evidence in the form of spectra which attests that the secondary phase or impurity formation did not take place during the hydrothermal processing of the materials. Furthermore, Figure 3 also validates that g-C_3_N_4_ and CdO co-evolved during the processing as their peculiar vibrational modes reported at the same positions [33,38,54], and the findings from XRD and SEM and HR-TEM characterization confirm the idea. Consequently, it can be surmised that the intermixing of CdO with g-C_3_N_4_ by following the specific conditions and parameters of hydrothermal treatment is feasible to fabricate crystalline nanorods of CdO-g-C_3_N_4_ composite.

### 2.4. UV-Visible Analysis

**Figure 4:**
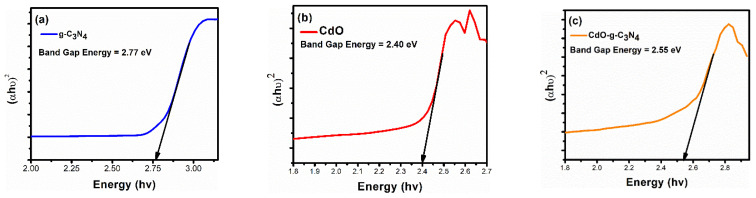
Tauc Plots of all the prepared materials, (**a**) pure g-C_3_N_4_, (**b**) CdO nanorods, and (**c**) CdO-g-C_3_N_4_ photocatalysts showing band gap energies (Eg). The band gap of the composite lies in between both of pure g-C_3_N_4_, CdO nanorods.

The optical properties, i.e., absorption spectrum and band gap energy of pure g-C_3_N_4_, CdO, and CdO-g-C_3_N_4_ composite are evaluated through UV-VIS absorption spectroscopy [55]. The UV absorbance data is used to formulate Tauc Plots [56] as shown in Figure 4. From the given Tauc Plots, band gap energy (E_g_) is determined [57,58]. Based on the calculations, it is revealed that the bandgap energies for g-C_3_N_4_, CdO & CdO-g-C_3_N_4_ are found to be 2.77 eV, 2.40 eV, and 2.55 eV, respectively. It is evident that intermixing of cadmium oxide (CdO, E_g_ = 2.40 eV) with graphitic carbon nitride (g-C_3_N_4_, E_g_ = 2.77 eV) resulted in the procurement of a mean band gap energy of 2.55 eV for CdO-g-C_3_N_4_ composite. Therefore, it can be deduced that the probe sonication, along with the hydrothermal processing, resulted in near-to-perfect impregnation of CdO-g-C_3_N_4_ composite. Moreover, the band gap energy (2.55 eV) of the CdO-g-C_3_N_4_ composite confirms its candidature to act as a promising photocatalyst.

### 2.5. Photoluminesence (PL) Spectroscopy

**Figure 5:**
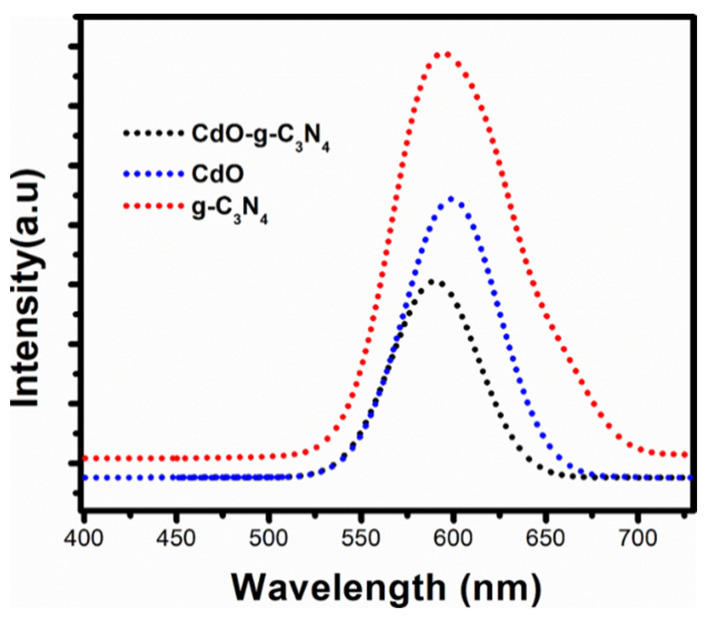
Photoluminescence spectroscopy of g-C_3_N_4_ nanosheets, CdO nanorods, and CdO-g-C_3_N_4_ nanocomposite.

Figure 5 shows the photoluminescence spectroscopy of g-C_3_N_4_, CdO, and CdO-g-C_3_N_4_ nanomaterials to study their charge carrier separation. The PL emission spectra was recorded at an excitation of 385 nm. The PL peaks maxima for each material centered approximately in the range of 590–599 nm [38]. It is observed from PL analysis that the peak intensity of g-C_3_N_4_ is significantly higher than that of its counterparts, i.e.,CdO and CdO-g-C_3_N_4_ nanocomposite, respectively. This indicates a fast recombination rate among the charge carriers in g-C_3_N_4_ nanosheets. Nevertheless, Figure 5 shows a declining PL intensity from CdO to CdO-g-C_3_N_4_. Hence, it is a supporting slower charge recombination in electron and hole pairs in the CdO-g-C_3_N_4_ photocatalyst [38]. The PL analysis further elaborate that rate of electrons hole pair recombination among charge carriers in g-C_3_N_4_ could be tuned by impregnation of metal oxides to form heteronanostructures. This confined recombination rate is beneficial to enhance the photoelectrochemical and photocatalytic activities of the nanocomposite materials for renewable and sustainable energy applications. Such enhanced PEC and photocatalytic characteristics are sufficiently observed in our prepared photocatalysts as discussed in the sections that follow.

## 3. Photoelectrochemical (PEC) Measurements

### 3.1. Chronoamperometric Analysis (CA)

**Figure 6:**
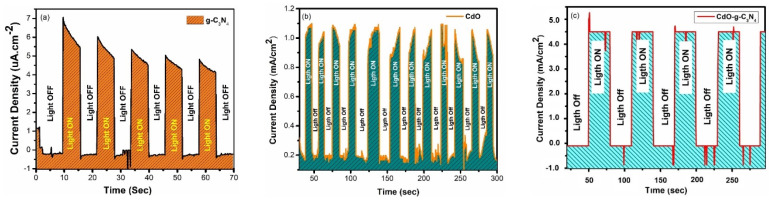
Chronoamperometric responses recorded at Open Circuit Potential (0 V applied potential) for (**a**) g-C_3_N_4_ nanosheets (**b**) CdO -nanorods and (**c**) CdO-g-C_3_N_4_ nanocomposite in 0.5 M NaSO_4_ electrolyte by a three-electrode system under chopped on/off light from a solar simulator of AM 1.5 G filter and 100 mW cm^−2^ (1 Sun) light intensity of Xe lamp.

The chronoamperometric (CA) results are displayed in Figure 6a–c for the pristine g-C_3_N_4_, CdO and doped CdO-g-C_3_N_4_ nanomaterials. In the CA outcome from fabricated nanomaterials, a photocurrent profile (*J_p_–t)* is shown, where photocurrent produced is plotted as a function of time under 1 Sun visible light exposure in a neutral electrolyte (0.5 M Na_2_SO_4_). In Figure 6a,b it is observed that the pristine g-C_3_N_4_ nanosheets and CdO nanorods generated photocurrent densities of 5 uA/cm^2^ and 800 µA/cm^2^ respectively. Conversely, in similar circumstances, CdO-g-C_3_N_4_ composite manifests a photocurrent density of 4.5 mA/cm^2^, and the photocurrent density is 500% more than the photocurrent density recorded for pristine g-C_3_N_4_ nanosheets and CdO nanorods. Based on the photocurrent densities of the fabricated materials, it can be inferred that the CdO-g-C_3_N_4_ composite produced better PEC outcomes than their counter parts (CdO and g-C_3_N_4_). Likewise, from Figure 6c it can be learned that the CdO-g-C_3_N_4_ composite continuously maintained the same amount of photocurrent upon exposure to 1 SUN irradiation in regular intervals under the same experimental conditions. Consistent behavior of the material under visible light shows sound adherence and stability of fabricated material layers on the FTO substrates. Moreover, the larger photocurrent density of the CdO-g-C_3_N_4_ composite also indicates that pre and post hydrothermal processing of the CdO-g-C_3_N_4_ composite played a vital role in its heterojunction formation and charge transferability among g-C_3_N_4_ & CdO has drastically amplified. Consequently, the two factors ensued enhanced photocatalytic activity of the CdO-g-C_3_N_4_ composite by inhibiting the recoupling of the electron-hole pairs.

### 3.2. Linear Sweep Voltammetry (LSV)

**Figure 7:**
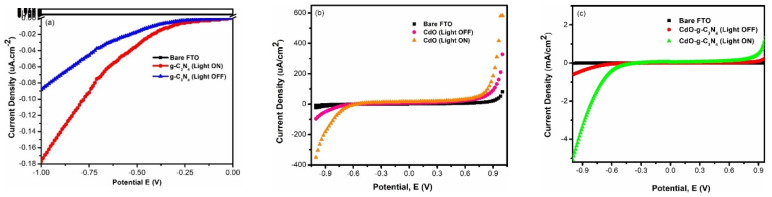
Linear Sweep Voltammetry profile in applied potential range of −1 V to 1 V, at a scan rate of 100 mV/s in 0.5 M Na_2_SO_4_ aqueous solution (**a**) g-C_3_N_4_ nanosheets (**b**) CdO -nanorods and (**c**) CdO-g-C_3_N_4_ nanocomposite in a three-electrode system under 1 Sun light irradiation in an OFF and ON light mode.

The linear sweep voltammetry was performed for g-C_3_N_4_, CdO, and composite CdO-g-C_3_N_4._ The results are shown in Figure 7a–c, respectively. In each figure, various trends were observed for bare FTO, pure g-C_3_N_4_, CdO, and composite CdO-g-C_3_N_4_, with and without the exposure to 1 SUN irradiation. It is evident from the graphs that the bare FTO substrates showed insignificant response to one SUN irradiation. In contrast, g-C_3_N_4_, CdO, registered mild responses in terms of photocurrent densities of 0.18 µA·cm^−2^ and 400 µA·cm^−2^ respectively at a scan rate of 100 mv/s in a potential range of 1 V to −1 V in 0.5 M Na_2_SO_4_ electrolyte. Under the same parameters, CdO-g-C_3_N_4_ nanorod composite performed outstandingly by displaying the highest value of photocurrent density 5 mA·cm^−2^. These results are in good agreement with the chronoamperometric (CA) measurements as presented in Figure 6. It is evident from Figure 7c that the photocurrent density of CdO-g-C_3_N_4_ improved drastically and thus induced a notable uncoupling of electron-hole pairs by offering a lesser amount of resistance rather than individual components g-C_3_N_4_ and CdO. Hence, the composite CdO-g-C_3_N_4_ offered significantly more hindrance in electron-hole rejoining. In addition, the artful impregnation of CdO and g-C_3_N_4_ through sonochemical treatment, followed by hydrothermal processing produced a synergistic effect by amplifying the photocatalytic properties of the CdO-g-C_3_N_4_ composite. Furthermore, the photocurrent densities of each material; g-C_3_N_4_ and CdO, and CdO-g-C_3_N_4_ are correlated with their capability to accommodate hydrogen evolution by reduction reactions and charge separation of photoexcited elections from holes.

### 3.3. Electrochemical Impedance Spectroscopic (EIS) Analysis

**Figure 8:**
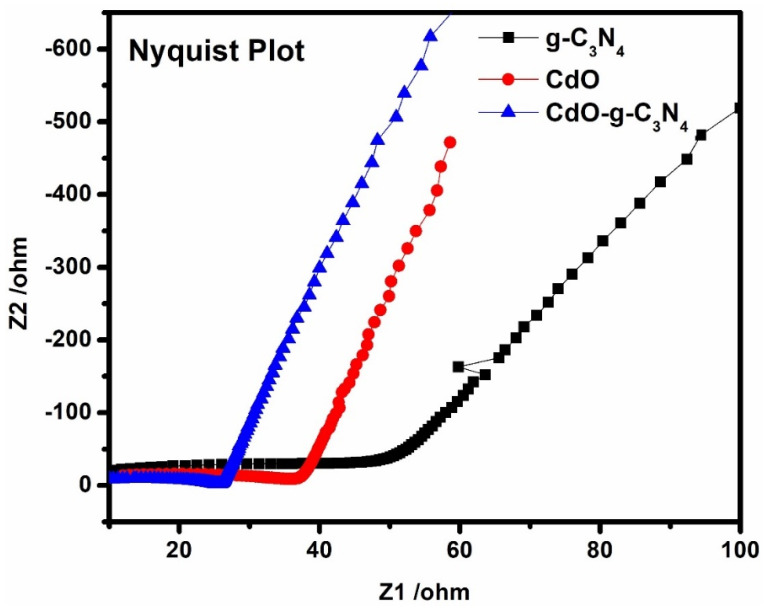
EIS analysis and Nyquist plot of g-C_3_N_4_, CdO and CdO-g-C_3_N_4_.

Electrochemical impedance spectroscopy (EIS) was conducted to determine the charge transfer resistance of the synthesized materials, i.e., g-C_3_N_4_, CdO, and CdO-g-C_3_N_4_. The EIS characterization was performed in 0.5 M Na_2_SO_4_ aqueous solution under visible light illumination in Constant E mode with frequency range of 15–300,000 Hz. As a result, the Nyquist plots were obtained for the synthesized materials under visible light exposure as displayed in Figure 8. It is noticed that all the synthesized materials showed a semicircle along with a straight-line slope under visible light. The semicircles are in the area of high frequency. Generally, the semicircle in the region of high frequency corresponds to the electron transfer processes. In contrast, the straight slope in the low-frequency region corresponds to the electrode’s ion diffusion. Therefore, the value of charge transfer resistance (*R_t_*) is directly proportional to the diameter of the semicircle. Figure 8 demonstrates that the arc diameter of CdO-g-C_3_N_4_ is lower than that of CdO and g-C_3_N_4_ EIS curves. Yet again, it confirmed that the sonochemical fabrication, followed by a hydrothermal approach, has improved the electrochemical conductivity of the CdO-g-C_3_N_4_ photocatalyst. Likewise, the straight slope of the CdO-g-C_3_N_4_ photocatalyst is higher than that of CdO and g-C_3_N_4,_ which supports the relatively enhanced ionic diffusion rate. Thus, EIS studies and the PEC outcomes as discussed above have revealed that enhanced photocurrent density, low resistance, improved charge transfer, formation of heterojunction, lower electrical resistance and higher ionic diffusion are the reasons for better photocatalytic efficiency of CdO-g-C_3_N_4_ photocatalyst that can generate hydrogen from an aqueous medium.

### 3.4. Charge Transfer Mechanism

**Figure 9:**
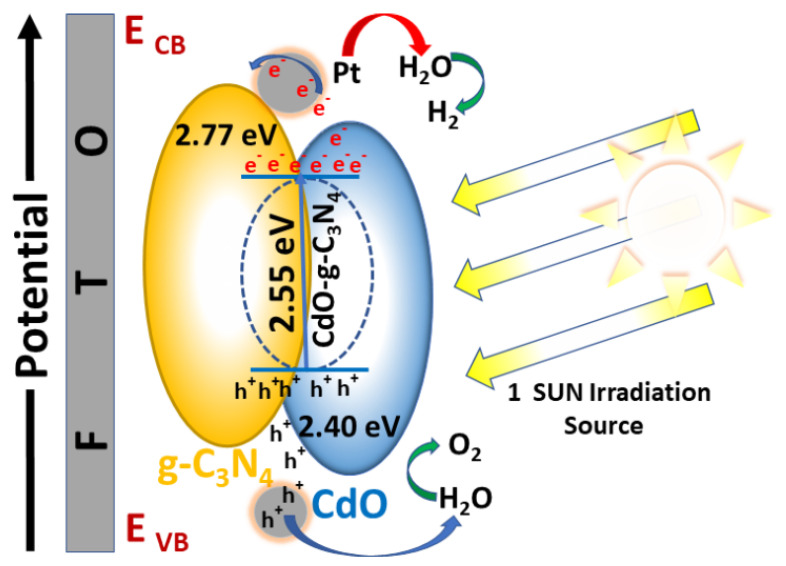
The proposed charge transfer mechanism by g-C_3_N_4_, CdO and CdO-g-C_3_N_4_ Photocatalyst.

The charge transfer diagram shown in Figure 9 also supports that the photocurrent densities correspond to oxidation (oxygen production) and reduction reactions (hydrogen generation). The increase in the electrochemical efficiency of the CdO-g-C_3_N_4_ photoanodes compared to CdO, and g-C_3_N_4_ photoanodes, is attributed to effective separation of electron-hole pairs and the low resistance offered by CdO-g-C_3_N_4_ compared to CdO and g-C_3_N_4,_ photocatalysts. Moreover, the highest photocurrent density by CdO-g-C_3_N_4_ is credited to the synergistic effect achieved by the sonochemical and hydrothermal fabrication of CdO with g-C_3_N_4_. The interaction between g-C_3_N_4_ and CdO in the CdO-g-C_3_N_4_ photocatalyst effectively reduced the charge transfer distance of photoexcited electrons and inhibited their further recombination with holes. Subsequently, this charge separation predominantly facilitated photocurrent generation and H_2_ evolution, thus showing enhanced PEC and photocatalytic characteristics of the CdO-g-C_3_N_4_ composite.

## 4. Experimental Strategies

### 4.1. Material and Chemicals

All the chemicals/solvents used (as received) during the experiments were of analytical grade and purchased from well-known companies/suppliers, i.e., Sigma Aldrich/Alfa Aesar. A local vendor was also contacted to obtain Fluorine-doped tin oxide (FTO) substrates of the following specific features: size (l × w × t = 25 × 25 × 1.1 mm), R (resistivity= 7–15 ohms) and transmittance efficiency of more than 80%. Before using the FTO substrates, the FTO substrates underwent ultrasonication treatment for 15 min in Deionized (DI) water and ethanol.

### 4.2. Synthesis of Graphitic-Carbon Nitride (g-C_3_N_4_)

The graphitic carbon nitride g-C_3_N_4_ was synthesized from melamine by following the steps already reported in the literature with some alterations [59,60,61]. In the beginning, melamine powder was weighed properly, and the known amount of the substance was subjected to heat treatment (annealing) in a muffle furnace at 550 °C for 300 min to finally obtain a yellow-colored bulk g-C_3_N_4_. To convert bulk g-C_3_N_4_ to nanosized sheets, the process underwent a physical transformation procedure [62]. Initially, the powder of bulk g-C_3_N_4_ was mixed with ethanol (70% sol.) using a probe sonication technique for 180 min. Then the solution was centrifuged (6000 rpm) to collect the desired nanosheets. Eventually, the g-C_3_N_4_ was dried at 150 °C in a vacuum oven for 120 min.

### 4.3. Synthesis of Cadmium Oxide (CdO)

The CdO nanoparticles were synthesized by chemical precipitation method with trivial changes as described by Li et al. [63] and Sumeet et al. [64]. In a typical chemical reaction, Cd (NO_3_)_2_·4H_2_O salt was put into the water (DI) to obtain a 0.5 M solution. Sodium hydroxide (1 M) was added to the solution in the form of droplets by using a magnetic stirrer for mixing until the solution became slightly basic (pH = 8). Meanwhile, adding NaOH to the solution, the CdO will precipitate out. The reaction was allowed to take place while continuously stirring at 60 °C for 80 min. In the next stage, an ultrasonication of the solution was performed at 60 °C for another hour. To collect the CdO precipitates from the solution, it was subjected to centrifuge for 20 min at 10,000 rpm. The obtained precipitates were washed several times using DI water and ethanol to remove the impurities and unreacted ingredients. Later, the precipitates obtained were dried in the oven at 100 °C for 12 h [34]. Subsequently, CdO nanorods were obtained after annealing at 700 °C for 4 h.

### 4.4. Synthesis of Cadmium Oxide Dopped Graphitic Carbon Nitride (CdO-g-C_3_N_4_) Nanocomposite

A hydrothermal method was adopted to produce CdO-g-C_3_N_4_ nanocomposite. Initially, two separate 0.1 molar (0.642 g of CdO & 0.460 g of g-C_3_N_4_ nanosheets) aqueous suspensions in 50 mL DI water were prepared by 1 h sonication. In the next step, to obtain a homogeneous mixture, the CdO and g-C_3_N_4_ aqueous solutions were mixed using sonication for another hour. After the sonication, the homogenous solution was transferred into PPL-lined vessels (stainless steel autoclave). A day-long (24 h) heat-treatment of the homogenous solution was performed in the autoclave at 180 °C. Later, the thermally heat treated homogenous aqueous solution was subjected to centrifugation at 6000 RPMs for 5 min, and the obtained CdO-g-C_3_N_4_ nanocomposite was washed using ethanol and DI water at least three times before being placed in the vacuum oven for further drying at 150 °C for 120 min. After the drying, the powdered CdO-g-C_3_N_4_ nanocomposite underwent annealing for a further 4 h at 550 °C.

## 5. Characterization

The manufactured materials were characterized using advanced characterization techniques; UV-VIS, FTIR, PL, XRD, FE-SEM and HR-TEM, etc. A 750-Watt probe sonicater (Model: Cole Parmer 500) was used to perform the sonochemical treatment. The powder obtained after the sonochemical procedure was further characterized by XRD to determine phase formation in the developed materials. The characterization was performed within the 2θ range of 10–80° with a step size of 5° min^−1^ (Model: XRD-6100 by SHIMADZU), and the X-Rays source was Cu Kα (wavelength of 1.5409 Å) operated at 80 mA/60 kV. The XRD pattern acquired was matched by the database of the ICDD-PDF-2 library and it confirmed the formation of g-C_3_N_4_, CdO and CdO-g-C_3_N_4_. The shape and morphology of the materials under investigation were observed using a scanning electron microscope TESCAN Lyra 3 Field Emission Dual Beam (Electron/Focused Ion Beam) system combined with a high-end field-emission scanning electron microscope (FE-SEM). The morphology at higher resolution and selected area diffracted pattern (SAED) from CdO-g-C_3_N_4_ nanocomposite were recorded on JEOL (JEM2100F) high resolution transmission electron microscopy (HRTEM) at 200 KV accelerating voltage. The optical characterizations of the developed materials (g-C_3_N_4_, CdO & CdO-g-C_3_N_4_) was performed by using a special UV-VIS spectrometer (Model: Jenway 6850 double beam with variable bandwidth) to measure the absorbance. The FTIR (SHIMADZU IRAffinity-1s) was used to conduct vibrational spectroscopic studies. A fluorolog-3 imaging spectrophotometer was used to measure the photoluminescence activity of the nanomaterials at an excitation wavelength of 385 nm and a slit width of 2 nm. The photoelectrochemical studies of the materials were carried out using a potentiostat (IVIUM-n-stat). The electrochemical workstation was equipped with multichannel electrochemical analysis. Solar Simulator (Model: ABET 10500) was used to generate 1 SUN visible light for photoelectrochemical studies. In addition, the solar simulator contained the specifications: AM 1.5 G output, UV-Cut of filter & D.C. Xenon arc lamp, and it was calibrated as per the standards ASTM, IEC & JIS class A.

## 6. Fabrication of g-C_3_N_4_/FTO, CdO/FTO, and CdO-g-C_3_N_4_/FTO Photoanodes for Photoelectrochemical (PEC) Studies

The slurries of the developed materials g-C_3_N_4_, CdO and CdO-g-C_3_N_4_ were coated over the conducting substrates of Fluorinated Tin Oxide glass (FTO) and were used for photoelectrochemical (PEC) studies. Before the coating, the FTO glass substrates were thoroughly cleaned using acetone and DI water in order, following the ultrasonication. The slurries were prepared separately by taking 100 mg of each material and blending in a mixture containing 70% ethanol, 30% DI water and a few drops of 5% Nafion. The FTO substrates that were pretreated by drop-casting were taken and a slurry of each prepared material was coated over the conducting side. Then, the coated slurry was exposed to 100 °C for 2 h to completely evaporate the ethanol and DI water from the films. After heat treatment, uniform layers/films of g-C_3_N_4_, CdO and CdO-g-C_3_N_4_ were obtained over the FTO substrates. These FTO substrates were subjected to photoelectrochemical (PEC) characterization using Ivium multichannel Potentiostat (Ivium-n-Stat) in a 3-electrode system in which the electrolyte used was sodium sulphate (0.5 M) with a neutral pH of 7. The platinum electrode (wired) was employed as a counter electrode, the prepared material films, i.e., g-C_3_N_4_, CdO, and CdO-g-C_3_N_4_ acted as working electrodes and the standard calomel electrode worked as a reference electrode. To generate solar light of the equivalent intensity of one SUN, a solar simulator 10500 was used.

## 7. Conclusions

Hydrothermal and sonochemical methods are successfully applied for the synthesis of CdO-g-C_3_N_4_ photocatalyst. The sonochemical fabrication of CdO with g-C_3_N_4_ yielded a highly effective photocatalyst for solar driven water splitting to produce hydrogen. All the nanomaterials were characterized using SEM, HR-TEM, XRD, UV-VIS, PL and FTIR techniques. The XRD confirmed the formation of g-C_3_N_4_ and CdO and CdO-g-C_3_N_4_ phases with good crystallinity. FTIR spectroscopy showed characteristic peaks of corresponding functional groups in all the nanomaterials. The scanning electron microscopy revealed the nanorod like structure and morphology of Cd-Og-C_3_N_4_. The HR-TEM micrographs of CdO-g-C_3_N_4_ further confirmed nanorods like structure with a diameter range of 50–60 nm with an average length of 2–3 μm, which makes its surface area huge, making its surface more active for electron and holes interaction. SAED patterns also revealed its polycrystalline in nature and crystals are placed at different orientations which can be deduced from the numerous bright dots. 

Photoelectrochemical studies showed excellent water degradation efficiency of the CdO-g-C_3_N_4_ photocatalyst for Hydrogen evolution under 1 SUN visible light illumination. The Linear sweep voltammetry measurements revealed that the enhanced current density of 5 mA·cm^−2^ by CdO-g-C_3_N_4_ is due to the effective separation of photogenerated electron-hole pairs and low resistance offered by the photocatalyst. Similarly, the chronoamperometric analysis revealed the significant increment in photocurrent attributed to synergism between CdO and g-C_3_N_4_ that lowered the electron-hole recombination while moving from VB to CB. 

In comparison, the electrochemical impedance spectroscopy exposed the lower electrical resistance and higher ionic diffusion for CdO-g-C_3_N_4_ photocatalyst. Thus, the EIS study is also augmenting PL analysis, where low charge recombination in electron and hole pairs for CdO-g-C_3_N_4_ are observed. In summary, the CdO-g-C_3_N_4_ photocatalyst is a potential photocatalyst for hydrogen generation.

## Figures and Tables

**Table 1:** A comparison of various recent Cd/CdO nanomaterials or their composites with g-C_3_N_4_ and others for Photoelectrochemical/Photodegradation applications.

#	Photocatalyst	Synthetic Strategy	Photoelectrochemical/ Photodegradation Study	Band Gap Energy (eV)	Current Density/Hydrogen/Oxygen Generation	Ref.
1	CdO cauliflower	Mechanochemical process followed by heating treatment	95%, 91.5%, and 98% photodegradation Congo red, Malachite green and Crystal violet	2.22	_	[41]
2	CdS/CdO	Co-precipitation	92% Photodegradation of the RhB	2.9	_	[42]
3	CdO–TiO_2_	sol–gel method	>70% Methylene blue (MB), Methyl orange (MO) and Rhodamine B (Rh-B) degradation efficiency	3.12–3.19	_	[43]
4	CdO–CdS	Electrochemical deposition	71.1% of photodegradation efficiency of MB.	2.25–2.29	2.6 mA/cm^2^	[44]
5	ZnO/CdO	Thermal decomposition	97.8% degradation efficiency for MB	2.99	_	[45]
6	g-C_3_N_4/_CdO	Chemical precipitation & self-assembly	96% rhodamine B(RhB) dye removal efficiency.	2.35	_	[38]
7	Cd-g-C_3_N_4_	Thermal polymerization	98.1% tetracycline (TC) degradation efficiency.	_	_	[46]
8	Cd-WO3 (CWC) and Cd-doped WO3@g-C_3_N_4_ (CWCC)	Ultrasonication, stirring & thermolysis	95.98% Photodegradation Efficiency	2.06 & 1.85	_	[47]
9	NiO/Cd/g-C_3_N_4_	Microwave-assisted	81.8% MB degradation efficiency	3.6		[48]
10	Cd-ZnO/g-C_3_N_4_	Co-precipitation & hydrothermal	>90% MB degradation efficiency	2.50		[17]
11	CdO-g-C_3_N_4_	Precipitation and Hydrothermal method	92% efficiency of MB degradation	2.55	>5 mA/cm^2^/ H_2_ generation	Current work

## Data Availability

Not applicable.
